# Automated MRI quantification of volumetric per-muscle fat fractions in the proximal leg of patients with muscular dystrophies

**DOI:** 10.3389/fneur.2023.1200727

**Published:** 2023-05-24

**Authors:** Lotte Huysmans, Bram De Wel, Kristl G. Claeys, Frederik Maes

**Affiliations:** ^1^Processing Speech and Images, Department of Electrical Engineering, KU Leuven, Leuven, Belgium; ^2^Medical Imaging Research Center, University Hospitals Leuven, Leuven, Belgium; ^3^Laboratory for Muscle Diseases and Neuropathies, Department of Neurosciences, KU Leuven, and Leuven Brain Institute, Leuven, Belgium; ^4^Department of Neurology, University Hospitals Leuven, Leuven, Belgium

**Keywords:** muscular dystrophies, quantitative MRI, fat fraction quantification, muscle segmentation, deep learning

## Abstract

Muscular dystrophies (MD) are a class of rare genetic diseases resulting in progressive muscle weakness affecting specific muscle groups, depending on the type of disease. Disease progression is characterized by the gradual replacement of muscle tissue by fat, which can be assessed with fat-sensitive magnetic resonance imaging (MRI) and objectively evaluated by quantifying the fat fraction percentage (FF%) per muscle. Volumetric quantification of fat replacement over the full 3D extent of each muscle is more precise and potentially more sensitive than 2D quantification in few selected slices only, but it requires an accurate 3D segmentation of each muscle individually, which is time consuming when this has to be performed manually for a large number of muscles. A reliable, largely automated approach for 3D muscle segmentation is thus needed to facilitate the adoption of fat fraction quantification as a measure of MD disease progression in clinical routine practice, but this is challenging due to the variable appearance of the images and the ambiguity in the discrimination of the contours of adjacent muscles, especially when the normal image contrast is affected and diminished by the fat replacement. To deal with these challenges, we used deep learning to train AI-models to segment the muscles in the proximal leg from knee to hip in Dixon MRI images of healthy subjects as well as patients with MD. We demonstrate state-of-the-art segmentation results of all 18 muscles individually in terms of overlap (Dice score, DSC) with the manual ground truth delineation for images of cases with low fat infiltration (mean overall FF%: 11.3%; mean DSC: 95.3% per image, 84.4–97.3% per muscle) as well as with medium and high fat infiltration (mean overall FF%: 44.3%; mean DSC: 89.0% per image, 70.8–94.5% per muscle). In addition, we demonstrate that the segmentation performance is largely invariant to the field of view of the MRI scan, is generalizable to patients with different types of MD and that the manual delineation effort to create the training set can be drastically reduced without significant loss of segmentation quality by delineating only a subset of the slices.

## 1. Introduction

**Figure 1:**
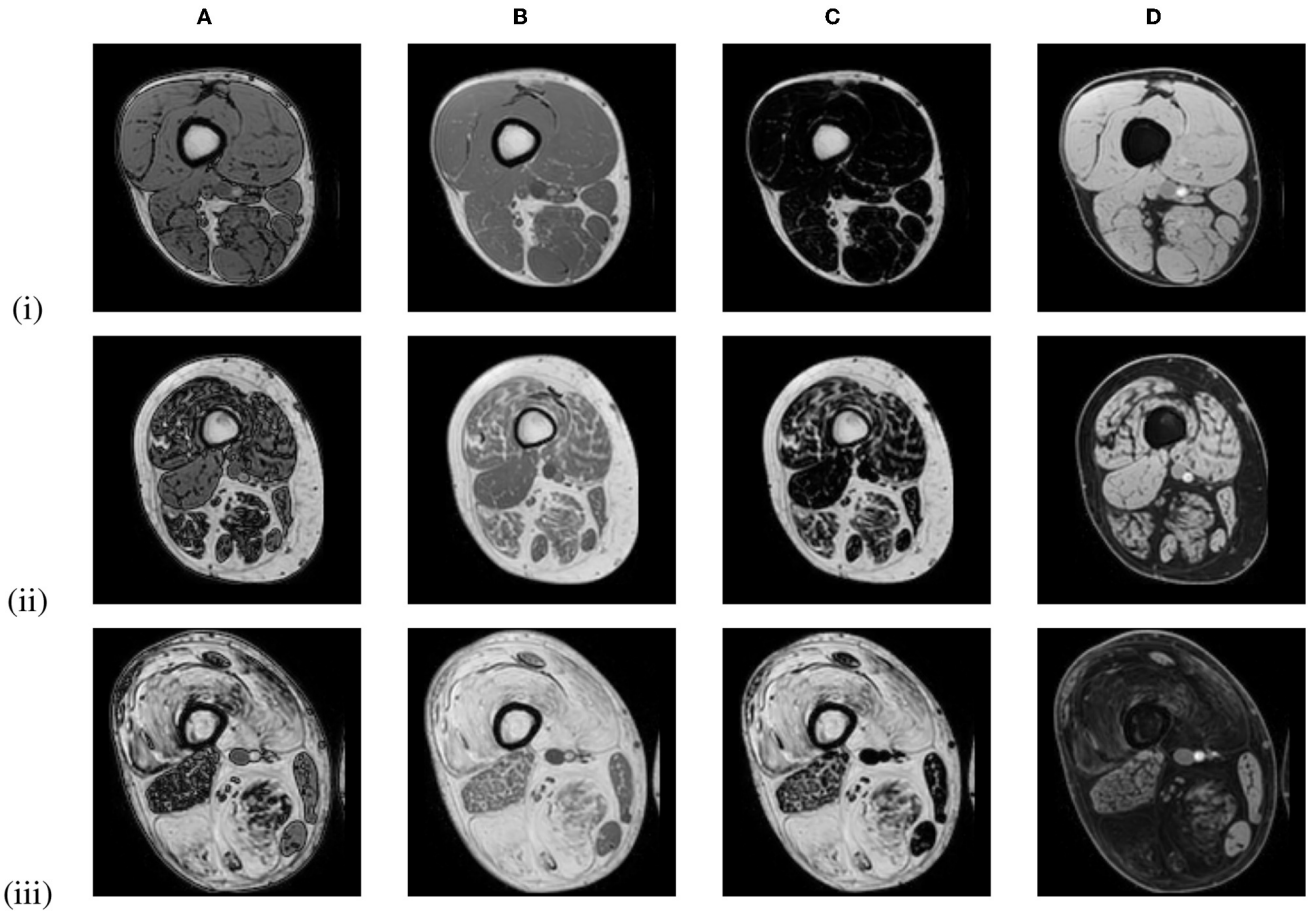
Out-of-phase **(A)**, in-phase **(B)**, fat-only **(C)**, and water-only **(D)** Dixon MRI images of the proximal leg of 3 LGMD patients in an early-stage (i), an intermediate-stage (ii) and an end-stage (iii) of the disease, showing increased replacement of muscle tissue by fat.

Limb-girdle muscular dystrophies (LGMD) are a group of rare genetic muscle diseases that mainly manifest in the proximal muscles around the hips and shoulders and that result in a gradual decline in muscle strength due to necrosis of muscle fibers and the replacement of muscle tissue by fat. Disease progression of patients with LGMD can be evaluated by assessing the degree of fat replacement in the muscles using fat-sensitive magnetic resonance imaging (MRI) of the proximal leg as illustrated in Figure 1, which can be objectively quantified by calculating the fat fraction percentage (FF%) within the muscles from these images (1). However, the FF% obtained by analysis of a single 2D slice is not reliable for assessment of disease progression as this implies the assumption of a homogeneous pattern of fat replacement along the proximodistal axis of the muscles, which is not the case for LGMD patients (1). Moreover, the degree of fat replacement may be different for different muscles. Hence, volumetric quantification of FF% for each muscle separately is to be preferred, for which an accurate 3D segmentation of each muscle individually in the MRI image volume is needed. As manual slice-by-slice delineation by a human expert is tedious and time consuming, in order to make volumetric quantification of FF% of individual muscles feasible in clinical practice, a reliable automated 3D muscle segmentation approach is required that is not only applicable to images of healthy subjects but it also sufficiently robust to perform accurately for images of patients with mild to severe fat replacement with large variation in image appearance.

The current state-of-the-art approaches for muscle segmentation still fall short of this requirement. Several approaches have been presented that consider the entire muscle region in the proximal leg as a whole, discriminating between healthy muscle tissue, inter-muscular and subcutaneous adipose tissue (2–6), or that segment entire muscle groups as a whole (7) instead of each muscle individually. These approaches only allow for global fat fraction analysis and the results are biased as not only inter-muscular fat but also fascia and blood vessels are included in the segmentation (8). While several approaches have also been presented for segmentation of individual muscles (9–15), most of these segment only part of the muscle in a limited field of view and not the entire muscle as in (12). Although promising results have been reported for muscle segmentation in healthy subjects, only few approaches were developed to be applied in patients with neuromuscular diseases (16). None of the mentioned approaches report segmentation performance for severely affected muscles except for (13), which reports good performance on mild and severely affected cases but uses a 2D approach that is trained on a limited amount of slices per patient. The interactive segmentation tool Dafne (15) is based on this work and uses a federated learning approach instead of the traditional training-validation-deployment technique which is used in this research.

This study presents and evaluates a clinically relevant approach for the automated 3D segmentation of 18 individual muscles of the proximal leg from knee to hip in healthy subjects and in subjects with MD and mild to severe fat infiltration, using deep learning models based on a 3D convolutional neural network (CNN) with U-Net architecture (17), which is currently the state-of-the-art for biomedical image segmentation (18). To deal with pathology, a separate model was first trained for healthy and mildly affected subjects and subsequently retrained and finetuned for more severe cases. We demonstrate the feasibility of quantifying FF% automatically in 3D in individual muscles over a broad range of per-muscle FF% values (4–92%) with clinically acceptable accuracy compared to manual analysis.

## 2. Materials and methods

### 2.1. Datasets

Two datasets of MRI scans of the proximal legs of patients with MD and of healthy controls were used in this work, acquired in two clinical studies. Written informed consent was obtained from all participants and the studies were approved by the Ethics Committee Research UZ/KU Leuven and performed in accordance with the relevant guidelines and regulations. The first dataset (LGMD dataset) consisted of scans of 29 LGMDR12 patients (19–70y) and 35 healthy control subjects (22–69y), acquired as part of a recently published study on LGMD characterization (1). The patients included 14 early-stage patients [total thigh FF% <20, corresponding roughly to Mercuri score (19) 0–1], and 15 intermediate- or end-stage patients (total thigh FF% >20, corresponding roughly to Mercuri score 2–4). For most subjects, two or three scans were available, acquired in different visits with about 1 year in between, resulting in 172 scans in total. The second dataset (BMD dataset) consisted of 42 scans of 21 Becker Muscular Dystrophy (BMD) patients, of which 7 early-stage and 14 intermediate- or end-stage patients, and 21 healthy control subjects. For each subject in this dataset, only a single scan was available.

All MRI scans were acquired with the same 1.5T MRI scanner (Philips Ingenia, Philips Medical Systems) using a 6-point Dixon 3D imaging sequence (20) with parameters: TR/TE/δTE = 9.2/1.36/1.3 ms, flip angle 12°, 140 slices, slice thickness 2 mm, interslice gap 0 mm, field of view (FOV) 450 × 394 × 280 mm, acquisition matrix 320 × 280 × 140. The images were reconstructed on a grid of 384 × 384 × 140 voxels with a voxel size of 1.2 × 1.2 × 2 mm. Figure 1 shows in-phase (IP), out-of-phase (OP), fat-only and water-only images of the proximal leg as obtained with this sequence for patients in different stages of muscle disease. The proton density fat fraction image was calculated as the fat-only image divided by the sum of the fat-only and water-only images and was used to quantify the FF% per muscle.

**Figure 2:**
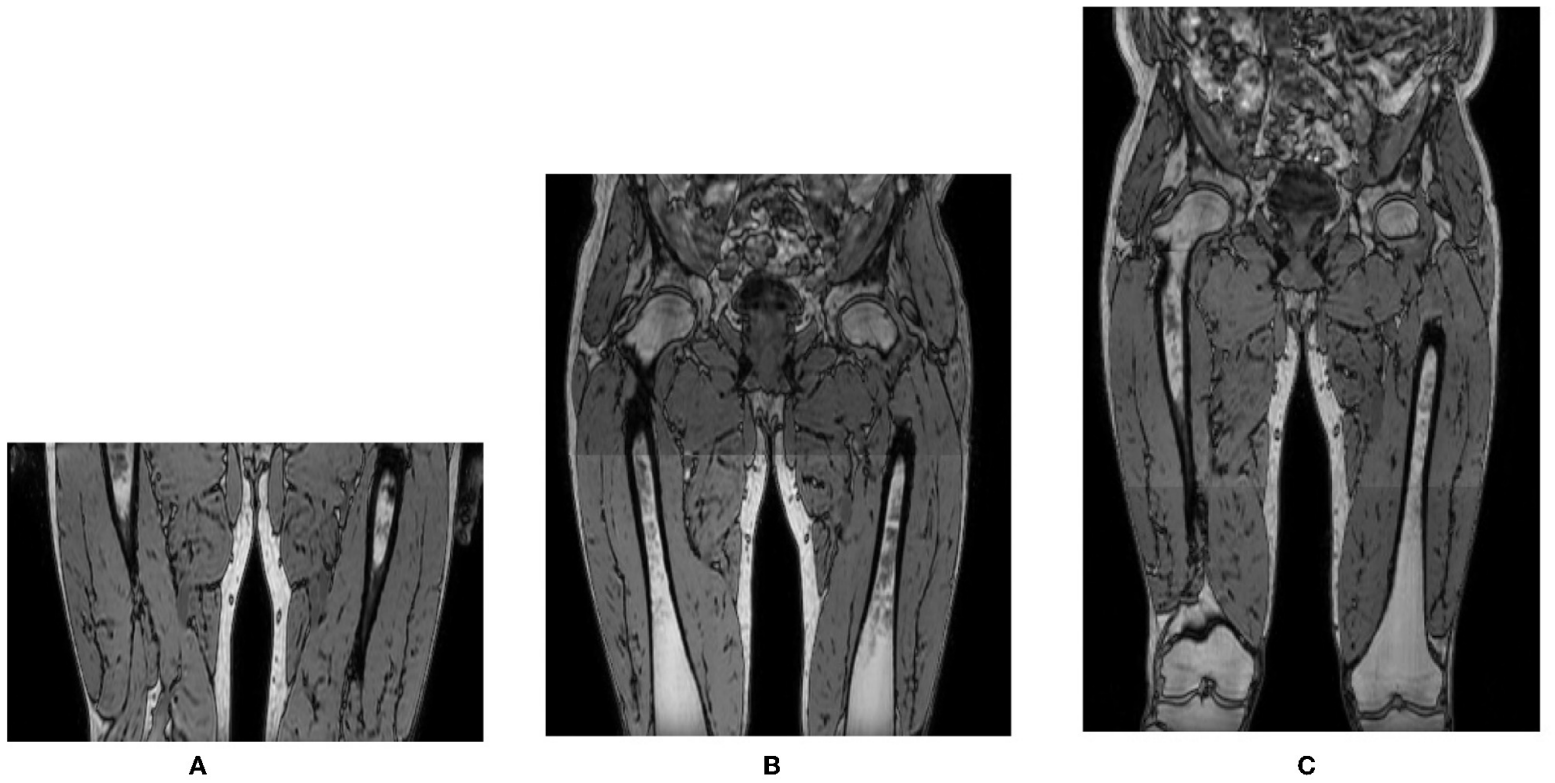
Out-of-phase images of the same subject acquired in different visits with 1, 2, or 3 overlapping 3D stacks of 140 slices with 2 mm slice spacing, covering a different inferior/superior extent of the proximal leg: **(A)** 1 stack, 140 slices; **(B)** 2 stacks, 270 slices; **(C)** 3 stacks, 355 slices.

Over the course of the LGMD study, the scan protocol changed from scanning a single stack of 140 MRI slices in the center of the proximal leg in visit 1 (V1), toward acquiring 2 and eventually 3 identical and partially overlapping such stacks in visit 2 (V2) and visit 3 (V3), covering the entire proximal leg from hip to knee. The stacks were stitched together to form one image volume. The LGMD dataset thus consisted of images with a different number of axial slices, covering a different inferior/superior extent of the proximal leg as illustrated in Figure 2. The scans of the BMD dataset were all acquired with 3 stacks, covering the entire proximal leg.

### 2.2. CNN architecture, training, and prediction

**Figure 3:**
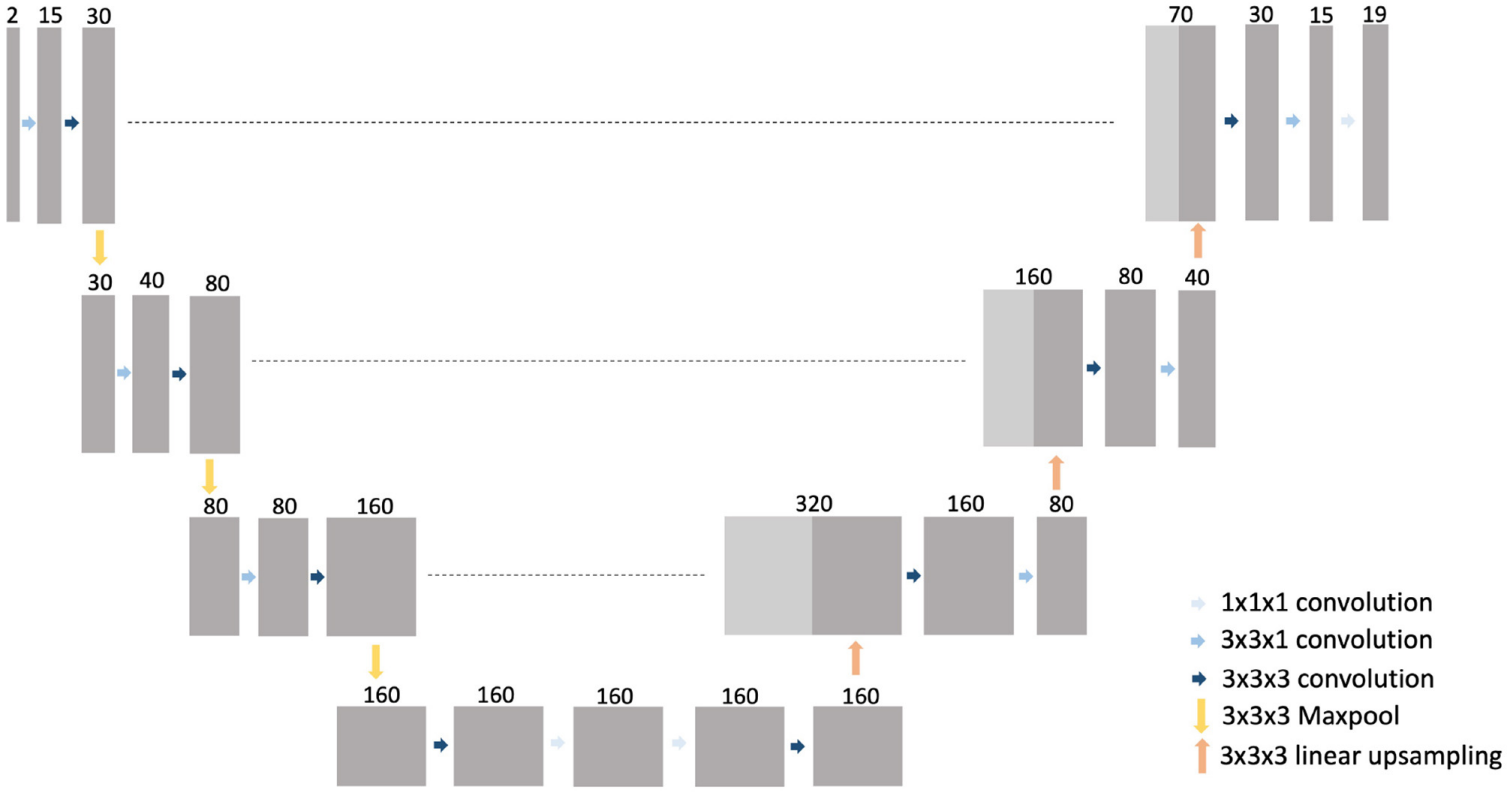
The 3D U-Net architecture of the trained CNN models.

A 3D CNN was trained to generate an automated segmentation of each of the 18 different muscles of the proximal leg individually, as listed in Table 1. The network architecture is depicted in Figure 3 and is based on the 3D U-Net presented in (21) with some minor changes: for down-sampling and up-sampling, a filter size of 3 × 3 × 3 was used and the deepest layer consisted out of 4 convolutional layers. The code is written in Python and makes use of the deepvoxnet2 package (https://github.com/JeroenBertels/deepvoxnet2) which uses Tensorflow and Keras (22).

**Table 1:** The 18 segmented muscles in the proximal leg.

**Rectus femoris (RF)**	**Biceps femoris caput brevis (BFB)**
Vastus lateralis (VL)	Semitendinosus (ST)
Vastus medialis (VM)	Semimembranosus (SM)
Vastus intermedius (VI)	Sartorius (SR)
Pectineus (PC)	Tensor fascia lata (TFL)
Adductor brevis (AB)	Gracilis (GC)
Adductor longus (AL)	Gluteus maximus (Gma)
Adductor magnus (AM)	Gluteus medius (Gme)
Biceps femoris caput longus (BFL)	Gluteus minimus (Gmi)

Both the OP and IP images were provided as input to the CNN. The original images were split in two equal halves along their mid-sagittal plane such that the left and right legs (and the left and right parts of the hip if present in the image) were separated. Symmetry between both legs was exploited by mirroring the left leg images to be similar to the right leg images. This simplified the problem to segmentation of the muscles in a single leg only as compared to both legs combined and doubled the amount of data available for training of the CNN. The input of the CNN consisted of a patch of size 2 × (192 × 384 × 81) voxels, containing a slab of 81 consecutive slices extracted from the single-leg OP and IP images, randomly positioned along the inferior/superior direction. By processing a patch instead of the entire volume at once, images with different amount of slices could be handled by the same CNN. The number of slices included in the patch was determined as a trade-off between maximal axial coverage and increasing memory requirements for training of the CNN.

During training, in every epoch 2 input patches were randomly sampled from each image in the training set. Standard data augmentation methods were applied to increase variation in the input data, such as affine transformation and adding of Gaussian noise. As 3D convolutions with a large patch size have a high memory consumption, the batch size was set to 1 in all experiments. Cross-entropy loss was used as loss function and Adam (23) was used as optimizer with an initial learning rate of 10^−4^. The learning rate was reduced with a factor of 2 when the validation loss did not improve over the last 25 epochs. The training ended after a fixed number of epochs or when the validation loss did no longer improve, as specified further.

A segmentation for an entire single-leg image volume was obtained by combining the output of the CNN for separate, partially overlapping patches randomly extracted from the volume. The prediction for the mirrored left leg input image was mirrored again to correctly represent a left leg and was stitched to the prediction of the corresponding right leg image. Connected component analysis was subsequently performed on the stitched volume to retain only the two largest, left and right leg components for each muscle, removing small blobs of incorrectly labeled voxels. Holes present inside a segmented muscle volume were filled subsequently.

### 2.3. Experimental setup

#### 2.3.1. LGMD dataset

**Figure 4:**
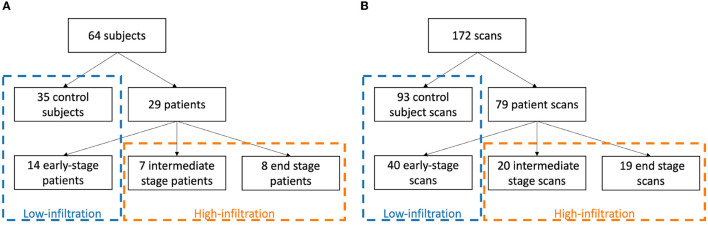
Division of subjects **(A)** and scans **(B)** in a low-infiltration and high-infiltration group according to clinical disease stage.

The LGMD dataset was used for training and testing of the deep learning models, based on precise manual delineations by a medical expert (BDW) of each muscle in both legs in every MRI slice of the original OP images (2 mm slice spacing) created using ITK-SNAP (24). To ensure that no fascia, intermuscular/subcutaneous fat or blood vessels were included, the segmentations were first checked by an experienced musculoskeletal radiologist and then a 1 pixel-wide layer was eroded from the boundary of every segmented muscle (1). To deal with the varying presence of pathology in the images, the dataset was split in a low-infiltration (LI) group, containing all scans of the control subjects and early-stage patients (49 subjects, 133 scans in total), and a high-infiltration (HI) group, containing the scans of the intermediate- and end-stage patients (15 subjects, 39 scans in total), (see Figure 4). Mean overall FF% and range of the scans in the LI and HI group was 11.3% [6.0–16.6%] and 44.3% [18.6–82.1%], respectively. Different CNN models were trained for the LI group and the HI group separately.

The scans of the LI group were randomly split in a training/validation set (35 subjects, 95 scans) and a test set (14 subjects, 38 scans). Splitting of scans was always performed such that all scans of the same patient were all included in the same subset. While including similar scans of the same subjects in the training set may increase the likelihood of overfitting, this is counteracted by the different field of view of the scans, the patch-based nature of the network and the use of a separate validation set during training. A five-fold cross validation approach was used to train 5 different CNN models on the training/validation set. The training of the LI models was terminated after a fixed number of 160 epochs. Each of these models was subsequently used to generate a prediction for the test set cases. An ensemble model was also created and evaluated on the test set by combining the outputs of the 5 individual models using a majority voting strategy. The best performing LI model, i.e., the model obtained in the fold that performed best on the LI test set, was selected and subsequently trained further on the images of the HI group to create a fine-tuned HI model. Again five-fold cross validation was performed to obtain different HI models, but no test set was separated due to the small number of subjects in the HI group. The training of the HI models was terminated after 500 epochs or when the validation loss did not improve over the last 40 epochs.

**Figure 5:**
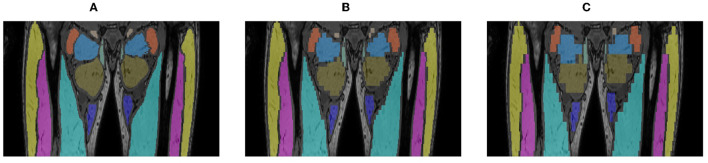
Alternative ground truth segmentations created by manual delineation of every slice [GT1, **(A)**], every 5th [GT5, **(B)**] or 10th slice [GT10, **(C)**], every 5th (GT5, **B**) or 10th slice (GT10, **C**), using nearest neighbor interpolation to fill in the intermediate slices.

The ground truth used to create the initial LI models was based on manual delineation of each muscle on every MRI slice (2 mm slice spacing). This is a tedious and time consuming process and especially complicated for muscles with high fat infiltration. To investigate whether the manual delineation effort could be reduced, two alternative ground truth segmentations were created by retaining the expert delineation on every 5th (10 mm spacing) or 10th (20 mm spacing) slice only and using nearest neighbor interpolation to fill in the segmentation for the slices in-between, as illustrated in Figure 5. Additional LI models were trained based on these simplified ground truths (GT5, GT10), using identically the same five-fold setup that was used for training the initial LI models based on the full ground truth (GT1). The performance of these different models was compared on the LI test set.

To investigate whether the different FOV of images acquired in different visits (V1, V2, V3) had an impact on the performance of the model, the segmentation accuracy of the LI GT1 ensemble model was evaluated separately on the 40 most distal, the 40 central and the 40 most proximal slices of the V1 scan and on the corresponding set of slices of the V2 and V3 scans of the same subject in the test set. To this end, corresponding slices between these different scans were identified manually by an expert (BDW). This analysis could be performed for 9 out of 14 subjects of the LI test set for which scans of all 3 visits were available and visually accurate correspondences between all considered slices could be established.

#### 2.3.2. BMD dataset

The generalizability of the LI and HI models trained on the LGMD dataset to images of other MD patients was examined by evaluating them on the BMD dataset. The best performing LI model was used to generate a segmentation for the images of the 21 healthy control subjects and 7 early-stage patients of the BMD dataset (mean FF%: 10.1%), while the best performing HI model was used to generate a segmentation for the images of the 14 intermediate- or end-stage patients of the BMD dataset (mean FF%: 58.4%). No independently obtained ground truth delineations were available for this dataset to assess accuracy of the automated segmentations. Instead, the segmentations generated by the model were visually verified by a medical expert (BDW) and corrected as needed. The accuracy of the LI and HI models on the BMD dataset was assessed by comparing the automated segmentations generated by the model with the manually corrected segmentations.

### 2.4. Evaluation

Agreement between the automated segmentations generated by the LI and HI models and the reference segmentations (the manual segmentations for the LGMD dataset, the corrected segmentations for the BMD dataset) was assessed using the Dice Similarity Coefficient (DSC) and the average symmetric surface distance (ASSD). DSC (in %) measures the volumetric overlap of two structures in an image by the ratio of the volume of their intersection over the mean of their individual volumes, yielding a value of 0% if there is no overlap between both and a value of 100% if there is perfect overlap. For a given image, DSC was either computed for each muscle separately (left and right leg combined) or for all muscles at once using the generalized DSC (25) to obtain a single measure per image. ASSD (in mm) measures the surface alignment of two structures by finding for each boundary point of either structure the closest point on the boundary of the other structure and averaging the distance between them over all such pairs. ASSD was computed for each muscle separately (left and right leg combined). Results are reported by the mean and standard deviation of DSC or ASSD over the entire validation or test set, and are calculated after the postprocessing step. The Wilcoxon signed-rank test was used to assess the statistical significance of differences in performance between different models (*p*-value smaller than 0.05).

## 3. Results

### 3.1. LGMD dataset

**Figure 6:**
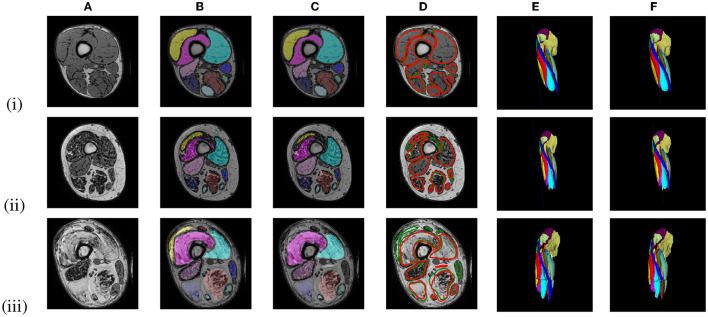
Out-of-phase **(A)** image with ground truth GT1 segmentation **(B, E)** and the U-Net prediction **(C, F)** of 3 LGMD patients in an early-stage (i), an intermediate-stage (ii) and end-stage (iii) of the disease. A comparison between the ground truth labels (green) and the predicted labels (red) is presented in **(D)**.

Figure 6 shows representative segmentation results for images of an early-stage patient obtained with the LI model, and of an intermediate- and an end-stage patient obtained with the HI model. Table 2 summarizes the validation and test set results for the LI models trained with different ground truths (GT1, GT5, GT10) and the validation set results for the HI models.

**Table 2:** Validation and test set results (*n* = number of cases) obtained by five-fold cross-validation for the LI and HI models with ground truth delineations defined on every slice (GT1), every 5th (GT5) or 10th slice (GT10).

	**LI model**						**HI model**	
	**Validation set**		**Test set**				**Validation set**	
	***n***	**GT1**	***n***	**GT1**	**GT5**	**GT10**	***n***	**GT1**
Fold 1	20	93.83 ± 2.18	38	94.78 ± 0.81	94.73 ± 0.85	94.31 ± 0.84	9	87.60 ± 3.03
Fold 2	17	94.33 ± 1.61	38	94.87 ± 0.77	94.58 ± 0.79	94.05 ± 0.93	9	90.90 ± 2.38
Fold 3	20	94.40 ± 1.20	38	94.78 ± 0.80	94.51 ± 0.90	94.20 ± 0.80	7	89.88 ± 3.51
Fold 4	18	94.10 ± 1.50	38	94.81 ± 0.83	94.66 ± 0.89	94.25 ± 0.85	7	90.86 ± 3.26
Fold 5	20	94.46 ± 1.09	38	94.94 ± 0.79	94.64 ± 0.94	94.22 ± 0.93	8	85.98 ± 1.43
Ensemble	-	-	38	95.27 ± 0.76	95.20 ± 0.76	94.90 ± 0.78	-	-

The ensemble test set results are obtained by combining the prediction outputs of the 5 individual models using a per voxel majority voting strategy.

None of the 5 individually trained LI models performed significantly differently on the test set than all others trained with the same ground truth, while their ensemble model performed better than any individual model. The difference in performance was statistically significant between the GT10 and GT1 ensemble models, but no significant difference was found between GT5 and GT1. The performance of the GT1 ensemble model was on average slightly better for the control subjects (mean DSC = 95.39 ± 0.68) than for the early-stage patients (mean DSC = 94.90 ± 0.89).

**Figure 7:**
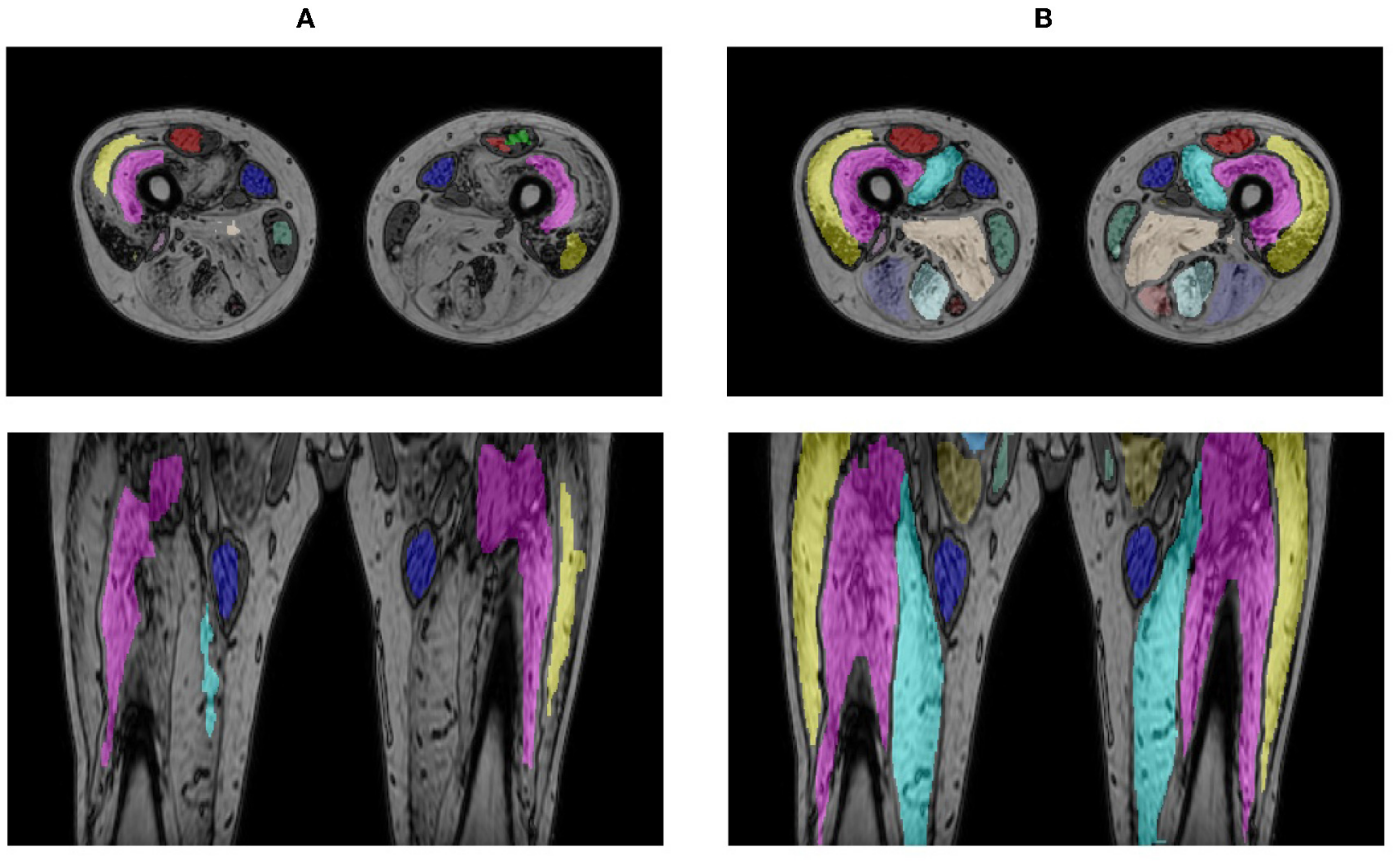
Segmentation result for an end-stage patient generated by the LI model **(A)** and the HI model **(B)**.

When applying the LI model that performed best on the LI test set (i.e., the model obtained for fold 5 in Table 2, DSC = 94.94) directly on the cases of the HI group, a mean DSC of 80.41 ± 20.54 and 49.13 ± 18.88 were obtained for the intermediate- and end-stage scans, respectively. A significant improvement in performance was observed for the fine-tuned HI models, with an average DSC of 90.03 ± 3.75 for the intermediate-stage patients and 87.83 ± 2.28 for the end-stage patients. Figure 7 shows the segmentation generated by the LI model (fold 5 LI GT1 in Table 2) and the segmentation generated by the HI model (fold 2 HI GT1 in Table 2) for an end-stage patient.

Table 3 summarizes the accuracy of the LI GT1 ensemble model evaluated in the same distal, central and proximal regions of the consecutive V1, V2, and V3 scans of the same subject. While no significant difference in segmentation performance was observed in the distal region, the accuracy was significantly better in the proximal region for the V3 scan with the largest FOV than for the V1 and V2 scans with smaller FOV.

**Table 3:** LI group test set results for the LI ensemble model applied to images of the same subjects with different FOV (V1, V2, V3) evaluated in corresponding regions defined in V1 (entire volume and distal, central, and proximal parts).

	***n***	**# slices**	**V1**	**V2**	**V3**
All	9	140	95.05 ± 0.60^+^	94.84 ± 0.61	95.84 ± 0.98
Distal	9	40	95.28 ± 1.09	95.21 ± 1.03	95.38 ± 3.42
Central	9	40	95.33 ± 0.62	94.04 ± 0.83^+^	96.09 ± 0.60
Proximal	9	40	94.46 ± 0.97^+^	94.29 ± 1.18^+^	95.75 ± 0.70

The + sign indicates a significant difference with V3.

**Figure 8:**
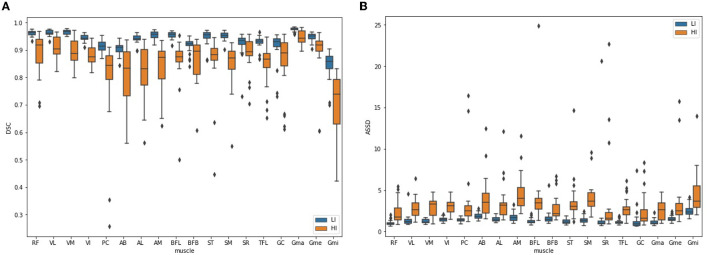
Boxplot of DSC **(A)** and ASSD **(B)** per muscle for the LI group (blue) and the HI group (orange).

Table 4 summarizes the mean DSC and ASSD per muscle for the LI group and the HI group separately, obtained by averaging over the LI test cases and the HI validation cases, respectively. Only the V2 and V3 scans were considered in this analysis: the V1 scans were omitted because of their small FOV, as several muscles were only for a small part or not at all included in these images. Figure 8 plots the DSC and ASSD per muscle for both groups.

**Table 4:** DSC and ASSD per muscle for the LI group (ensemble model applied to the test set) and the HI group (validation set results) (*n* = number of cases, mean ± std).

**Muscles**	**LI group**			**HI group**		
	**Test set**			**Validation set**		
	***n***	**DSC (%)**	**ASSD (mm)**	***n***	**DSC (%)**	**ASSD (mm)**
RF	26	96.14 ± 0.97	1.02 ± 0.32	25	89.30 ± 7.46	2.26 ± 1.30
VL	26	96.18 ± 1.05	1.34 ± 0.70	25	90.83 ± 4.03	2.79 ± 1.20
VM	26	96.39 ± 0.81	1.24 ± 0.25	25	89.43 ± 4.54	3.02 ± 1.16
VI	26	94.36 ± 1.24	1.46 ± 0.28	25	88.18 ± 3.57	3.03 ± 0.94
PC	26	91.19 ± 2.25.	1.39 ± 0.27	25	79.15 ± 15.63	3.54 ± 3.75
AB	26	90.50 ± 2.14	1.88 ± 0.35	25	80.31 ± 10.56	3.96 ± 2.47
AL	26	94.12 ± 1.61	1.46 ± 0.28	25	82.11 ± 9.74	3.47 ± 2.27
AM	26	95.28 ± 1.53	1.73 ± 0.55	25	83.83 ± 8.35	4.63 ± 2.19
BFL	26	95.41 ± 1.24	1.23 ± 0.33	25	86.12 ± 8.48	4.15 ± 4.41
BFB	26	91.66 ± 2.68	1.65 ± 0.87	25	86.38 ± 7.66	2.75 ± 1.48
ST	26	94.64 ± 2.73	1.23 ± 0.33	25	86.01 ± 10.37	3.64 ± 2.57
SM	26	95.05 ± 1.40	1.39 ± 0.40	25	85.15 ± 7.80	4.05 ± 1.81
SR	26	92.24 ± 4.41	1.80 ± 3.85	25	88.54 ± 5.95	3.33 ± 5.00
TFL	26	92.67 ± 2.25	1.14 ± 0.27	25	84.60 ± 7.35	2.83 ± 1.28
GC	26	91.08 ± 5.75	1.33 ± 1.37	25	85.58 ± 10.35	2.50 ± 2.03
Gma	26	97.37 ± 0.74	1.13 ± 0.34	25	94.49 ± 2.42	2.63 ± 1.15
Gme	26	94.59 ± 1.50	1.54 ± 0.35	25	89.23 ± 8.97	3.51 ± 3.45
Gmi	26	84.44 ± 5.10	2.44 ± 0.63	25	70.84 ± 10.88	4.64 ± 2.67

**Figure 9:**
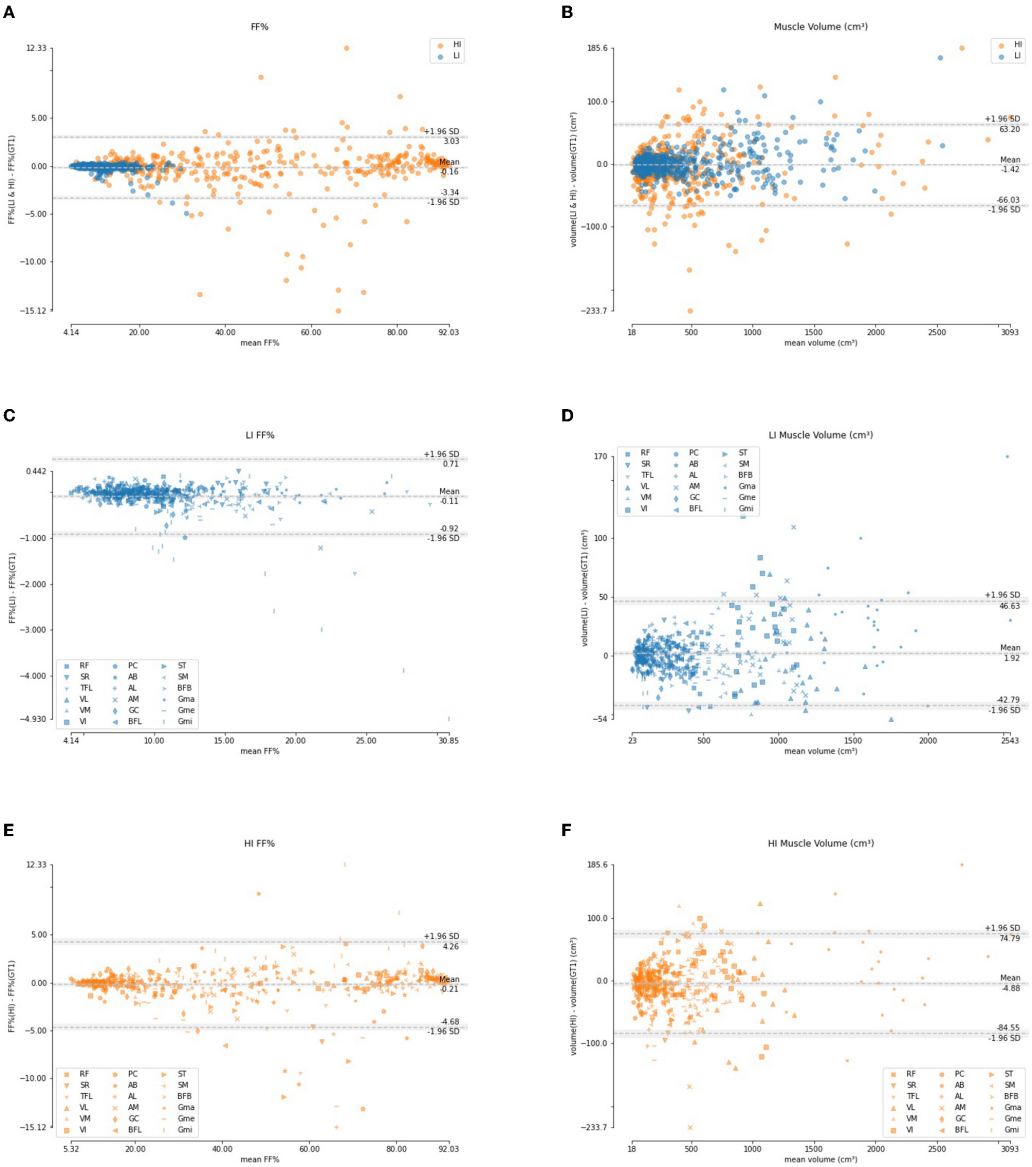
Bland-Altman analysis of the FF% per muscle **(A, C, E)** and muscle volume **(B, D, F)** for the LI group (blue) and the HI group (orange).

Figure 9 shows the Bland-Altman analysis for the quantification of FF% and muscle volume on these LI and HI cases. The FF% per muscle obtained using the automated segmentation agrees well with the FF% obtained using the manual ground truth delineation. For the LI group, the difference is mostly smaller than 1%, except for few outliers which are mainly due to the Gmi muscle, which was also an outlier for segmentation accuracy in Table 4. For the HI group, the difference shows a larger spread and more outliers, but the offset is still mostly limited to less than 5%. The difference in muscle volume between the manual and the automated segmentation tends to increase with larger muscle volume.

### 3.2. BMD dataset

**Figure 10:**
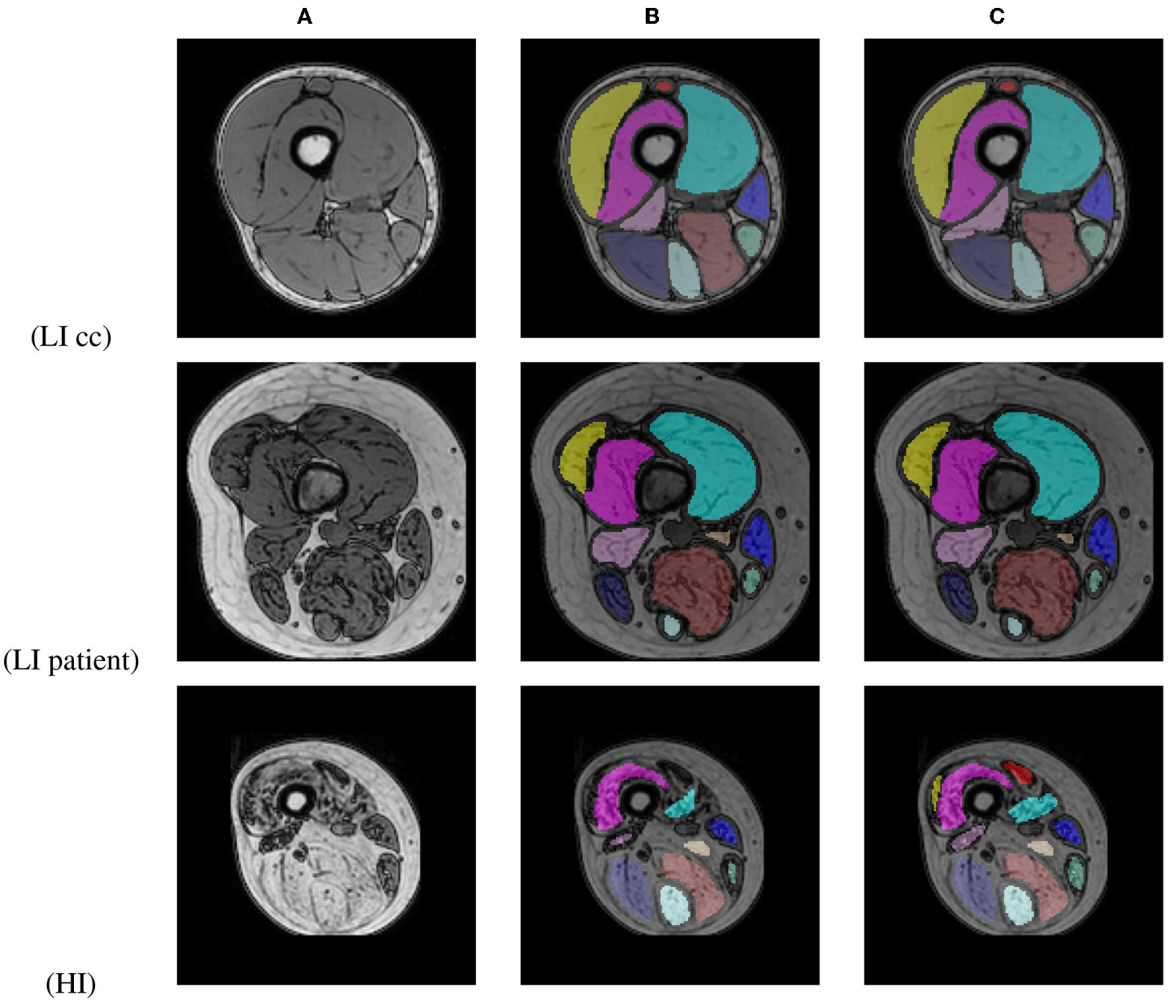
Out-of-phase image **(A)** with the U-Net prediction **(B)** and the manual corrected segmentation **(C)** of 2 BMD patients and one BMD control case.

An average per-image DSC of 98.31 ± 2.13 for LI cases (99.35 ± 0.55 for healthy control cases and 95.18 ± 2.05 for early-stage BMD patients) and 86.96 ± 21.24 for HI cases was obtained between the original and corrected segmentations. Figure 10 shows segmentation results for images of a BMD control case and an early-stage BMD patient obtained with the LI model, and of an end-stage BMD patient obtained with the HI model.

## 4. Discussion

The results in this paper show that a well trained U-Net can produce state-of-the-art results in the 3D segmentation of all 18 muscles individually in the proximal leg, knee to hip, for both healthy subjects and patients with muscle fat replacement. Bland-Altman analysis shows that the fat fraction per muscle derived from the automated segmentation matches well with the fat fraction calculated using the ground truth manual segmentations. The automatic segmentation approach is tested for robustness and the performance is found to be largely invariant with respect to the axial extent of the images, covering the entire proximal leg or only its central part and it generalizes well to new unseen data of patients with another muscular dystophy (Becker Muscular Dystrophy). Lastly the manual delineation effort to create the training set can be reduced by delineating every 5th slice (10 mm slice spacing) instead of every slice (2 mm slice spacing) without significant loss of segmentation quality. These AI-models for 3D muscle segmentation are thus instrumental toward the development and deployment of effective clinical tools for volumetric fat fraction quantification for the assessment of MD progression, which is increasingly important in natural history studies and clinical trials with novel treatments.

Rohm et al. (10) trained a U-Net to segment muscle groups in both healthy subjects and patients with a variety of neuromuscular disorders that show fatty infiltration in the muscles and succeeded in obtaining an average DSC of 85% for the healthy subjects and 80% for a dataset containing both healthy subjects and patients. Although our solution segments individual muscles instead of muscle groups, it outperforms the results obtained by Rohm et al. In both (9) and (11) DSC scores per muscle are reported. In (11) patients with fatty infiltrated muscles are included but with an average FF% much lower than the patients included in the HI group in our study. The DSC scores and ASSD values reported in (12) are similar compared to the ones obtained in this study for the LI group but their method uses a separately trained U-Net for every muscle. Our study succeeded in obtaining similar performance using a single U-Net in healthy subjects while segmenting entire muscles from knee to hip (FOV of 71.5 cm), and also reporting segmentation accuracy for mild to heavily infiltrated patients.

The DSC per muscle (Table 4 and Figure 8) shows a larger spread in segmentation performance and more outliers for the HI group than for the LI group, which makes sense because the muscle borders are less visible due to more fat infiltration. The muscles that on average had the largest FF% (SM, BFL, AM, AL, and Gmi) are also the muscles for which the largest differences in segmentation performance were observed between LI and HI models. Most of the outliers in the Bland-Altman analysis of FF% per muscle in the HI group (Figure 9E) originate from the scans of the 2 subjects with the largest mean FF% in our study (82 and 76%, respectively). The segmentation performance of Gmi in particular is significantly lower compared to the other muscles. The Gmi is a muscle in the hip region; therefore it was not present in the V1 images in our study, resulting in less training data for this muscle. If present in the images, it was located more at the periphery of the acquired MRI stack, where there are typically more artifacts. Certain parts of the Gmi are also easily infiltrated with fat and are hard to differentiate from subcutaneous fat, which could have lead to more variability in the ground truth segmentations.

A limitation is that due to the limited amount of scans available for severely affected LGMD patients no separate test set for the HI group was considered. When more training data would be available for this group it would be interesting to investigate whether similar performance can be obtained for severely fat infiltrated cases as for healthy subjects and whether a single U-Net CNN could be trained to handle both LI and HI cases. The HI model shows good performance for the independent BMD HI cases (average DSC of 86.96%), but as no a priori ground truth data is available for these cases, DSC may be positively biased as it is based on a correction of the model output.

Manual delineation as used in this work to generate the ground truth segmentations may be subject to inter-observer variability. Due to the time-consuming nature of manually delineating the individual muscles in the MRI stacks, it was not possible to have a second expert delineate all cases to generate a stronger ground truth. However, the same dataset was used in the study of De Wel et al. (1) where inter-observer variability was tested on a small subset of 3 images that were delineated independently by 2 observers, showing excellent manual segmentation reproducibility as quantified by the intra-class correlation coefficient.

The DSC scores for the HI BMD cases were slightly lower than the DSC scores obtained for the HI LGMD cases, mainly because of two outliers (DSC of 31 and 45%). We noted that both outliers in the BMD dataset suffered from severe atrophy in some of the muscles, especially in the quadriceps group. The model did not encounter this to such an extent within the LGMD dataset used for training and thus had more trouble with detecting the correct muscle borders for those two cases.

Postprocessing was applied to the initial model prediction to remove isolated blobs of segmented voxels and fill holes with unsegmented voxels. This resulted in an overall small average improvement in DSC of 0.02% for the LGMD LI test set and 0.1% for the HI validation set. Hence, the postprocessing does not result in a considerable improvement in segmentation performance, but reduces the time needed to correct the segmentations by removing small floating blobs and closing small holes.

As all scans were acquired using the same protocol on the same MRI scanner, no intensity normalization was applied between scans of different subjects. All models were thus trained using the original image intensity values without normalization. The drawback of using models that are trained on non-normalized MRI data is that they will not generalize well to images acquired with another MRI scanner or another scan protocol. Applying normalization during stitching of the separate stacks or to the stitched image using, e.g., z-score normalization, bias field correction or histogram equalization could be subject for future research.

## Data availability statement

The best performing LI (fold 5) and HI (fold 2) models together with example Python code and data are released to allow reproduction of the results and encourage further research (https://github.com/LHuysmans/3d-muscle-segmentation). The anonymized supporting datasets analyzed during the current study are available to qualified investigators from the corresponding author on reasonable request. Requests to access the datasets should be directed to lotte.huysmans@kuleuven.be.

## Ethics statement

The studies involving human participants were reviewed and approved by Ethics Committee Research UZ/KU Leuven. The patients/participants provided their written informed consent to participate in this study.

## Author contributions

LH developed and trained the neural networks, executed the experiments, summarized the results, and wrote the article. BD created the ground truth segmentations and reviewed the article. KC reviewed the article. FM reviewed and edited the article. All authors read and approved the final manuscript.
